# When resources run short: the inhibitory effect of economic scarcity on third-party punishment

**DOI:** 10.3389/fpsyg.2026.1801512

**Published:** 2026-04-22

**Authors:** Hanyu Liang, Jie Li

**Affiliations:** 1 Department of Psychology, Inner Mongolia Normal University, Hohhot, China; 2 Department of Psychology, Tianjin University of Traditional Chinese Medicine, Tianjin, China

**Keywords:** college students, economic scarcity, experimental study, longitudinal study, third-party punishment

## Abstract

This study investigates how economic scarcity influences third-party punishment, a costly and non-self-interested behavior that plays a critical role in maintaining social norms and cooperation. In real-world contexts, individuals often operate under resource constraints that may systematically alter their cost–benefit calculations; however, it remains unclear whether and how scarcity affects the willingness to punish unfairness. Across two experiments, we examined whether scarcity reduces individuals’ propensity to engage in third-party punishment. Experiment 1, involving 70 participants, showed that individuals under scarcity allocated significantly fewer resources to sanction unfair dictators than those in a control condition. Experiment 2, with 41 participants, replicated this design across two time points separated by 2 months. The results further confirmed the suppressing effect of scarcity on punishment and demonstrated a similar pattern of results across time points. These findings offer important theoretical and practical implications for promoting cooperation and effective norm enforcement under resource-limited conditions.

## 1 Introduction

Third-party punishment describes instances in which individuals willingly incur personal costs to sanction norm violators, even when the transgression poses no direct harm to themselves (Fehr and Fischbacher, 2004). As a distinctive manifestation of prosocial behavior, it involves a deliberate sacrifice of personal resources to uphold collective moral standards. This form of punishment reflects a moral imperative to preserve justice and constitutes a fundamental mechanism supporting social order and cooperative behavior (Fehr and Schurtenberger, 2018). Accordingly, it has attracted significant attention across psychology, behavioral economics, and cognitive neuroscience.

Extant research has systematically documented the psychological and contextual determinants of third-party punishment, highlighting the influences of emotional processes, individual differences, and situational cues (Marshall and McAuliffe, 2024; Martínez and Maner, 2024; White and Van De Vondervoort, 2025). Yet beyond establishing that such punitive behavior exists, a more fundamental question remains: who is inclined to intervene in norm violations and under what conditions. Several theoretical perspectives suggest that individuals may differ in their motivation to enforce social norms. From a norm-theoretic perspective, individuals vary in their sensitivity to social norms, and those who have more strongly internalized norms are generally more willing to incur personal costs to sanction violations (Bicchieri, 2017). However, such punishment behavior may not be driven solely by norm enforcement motives. Prior research suggests that, particularly under competitive or resource-constrained conditions, individuals’ punitive decisions may also reflect self-regarding considerations (e.g., Herrmann et al., 2008). This indicates that third-party punishment, while often serving a norm-enforcing function, may be influenced by a combination of normative and self-interested motivations. Against this backdrop, an important question concerns how situational factors shape individuals’ willingness to enforce social norms. The present research therefore focuses on how perceived economic scarcity affects individuals’ engagement in third-party punishment.

Building on this perspective, we argue that perceived economic scarcity may reduce individuals’ willingness to engage in third-party punishment because it prioritizes self-resource preservation. Under conditions of scarcity, individuals become more motivated to conserve their limited resources, making them less inclined to incur personal costs to sanction norm violations. In particular, in the context of widening global inequality and intensifying social stratification, individuals have become increasingly attuned to their own resource constraints (Auger et al., 2024). Perceived economic scarcity not only shapes decision-making under risk and interpersonal trust but may also fundamentally condition individuals’ sensitivity to issues of fairness and justice (Chen et al., 2023). Thus, economic scarcity may constitute a critical social-psychological factor underlying variability in third-party punishment, warranting systematic empirical investigation.

### 1.1 Economic scarcity and third-party punishment

Economic scarcity refers to the subjective psychological experience in which individuals perceive their available economic resources as insufficient to meet their needs (Haushofer and Fehr, 2014). Importantly, this concept is not confined to economically disadvantaged groups (Auger et al., 2024). Individuals across diverse social backgrounds may experience scarcity at different stages of life. Specifically, even those with relatively high incomes may perceive financial insufficiency due to heavy obligations such as mortgages, car loans, or education expenses. Moreover, rising living costs and intensified social comparison can exacerbate such perceptions among middle and high-income groups striving to maintain or enhance their socioeconomic status. Individual financial burdens and broader societal pressures may both generate persistent perceptions of scarcity. Such perceptions can arise even when income levels are objectively sufficient or above average. Thus, economic scarcity should be understood primarily as a subjective perception of resource deficiency rather than merely an objective economic condition (Shah et al., 2015).

According to the theory of economic scarcity (Shah et al., 2015), prolonged exposure to scarcity increases the likelihood of developing a scarcity mindset. Such a mindset narrows attentional focus toward the acquisition of pressing resources, thereby influencing attentional allocation and behavioral decision-making (De Bruijn and Antonides, 2022). This cognitive-behavioral pattern has been identified as a key psychological mechanism underlying maladaptive outcomes (Caballero et al., 2023; Seely et al., 2023). Within this framework, it is reasonable to hypothesize that economic scarcity may suppress individuals’ engagement in third-party punishment.

Although direct empirical evidence on the relationship between economic scarcity and third-party punishment remains limited, existing indirect evidence indicates a potential, though not unequivocal, association. For example, resource scarcity have been shown to activate a competitive orientation that prioritizes individuals’ own welfare (Roux et al., 2015). Similarly, perceptions of scarcity have been linked to reduced prosocial tendencies and a lower willingness to help others (Sun et al., 2024). Such findings suggest that scarcity may shift individuals’ attention and motivation toward the preservation of their own limited resources, potentially reducing their willingness to incur personal costs for others. Neuroscientific evidence provides additional support: experimentally induced scarcity reduces empathic responses to others’ suffering (Li et al., 2023), and empathy is widely recognized as a central driver of prosocial actions, including third-party punishment. Importantly, such effects appear to emerge primarily when norm violations directly threaten individuals’ access to critical resources (Luo et al., 2025). In contrast, third-party punishment paradigms typically involve situations in which norm enforcement entails personal costs without direct material benefits, making them especially sensitive to scarcity-induced self-protective motivations. This indicates the potential for nuanced or context-dependent effects, a gap our study seeks to clarify.

However, it is important to note that economic scarcity and broader socioeconomic disadvantage do not necessarily have uniformly negative effects on individuals. Psychological research comparing different socioeconomic status groups has found that individuals from lower socioeconomic status backgrounds sometimes display relative advantages in certain psychological traits. For example, in response to prolonged stress or life difficulties, lower SES individuals often show greater resilience and adaptability, with stronger interpersonal orientations that emphasize interdependence and social connection (Yang et al., 2026). These findings highlight the complexity of the relationship between economic scarcity and behavior.

### 1.2 Current study

To address this gap, the present study examines whether economic scarcity reduces individuals’ propensity to engage in third-party punishment. Two experiments were conducted. Experiment 1 used a within-subjects design to manipulate economic scarcity and assess corresponding changes in third-party punishment, thereby strengthening causal inference. Experiment 2 adopted a longitudinal design to explore the suppressive effect of economic scarcity on third-party punishment shows a similar pattern across time. Observing effects at a single time point offers limited insight into whether the suppression of third-party punishment persists or is influenced by temporal or situational factors. Experiment 2 goes beyond simple replication by assessing the same participants at two time points, spaced 2 months apart. This design allows us to examine the consistency of the effect of economic scarcity across time points, providing preliminary evidence regarding its potential temporal pattern.

The within-subjects design was chosen for several reasons. First, this design allows for the control of individual differences that might otherwise confound the results. By having each participant experience both scarcity and control conditions, we eliminate the potential variability in baseline tendencies (e.g., personal punishment levels or cognitive biases) that may influence third-party punishment. This increases the internal validity of our findings, as any observed differences can be attributed more confidently to the manipulation of scarcity rather than pre-existing individual differences. Furthermore, the within-subjects design increases statistical power, as each participant serves as their own control, leading to more precise estimates of the scarcity effect on third-party punishment.

Based on these considerations, we propose the following hypotheses:

*H1*: Economic scarcity reduces individuals’ propensity to engage in third-party punishment.

*H2*: The suppressing effect of economic scarcity on third-party punishment exhibits a similar pattern across time.

By linking economic scarcity to third-party punishment, this study integrates literatures on scarcity and norm enforcement, thereby extending both domains. By testing these hypotheses, this study aims to advance the understanding of the psychosocial functions of scarcity and provide theoretical implications for interventions designed to strengthen mechanisms of norm enforcement.

## 2 Method

### 2.1 Experiment 1

#### 2.1.1 Participants

Sample size was determined using G*Power 3.1. With a medium effect size (*f* = 0.25), *α* = 0.05, and power (1-*β*) = 0.95, the required sample was 23 participants. To account for potential attrition, 70 university students were randomly recruited (46 females; *M_age_* = 20.46 years, *SD* = 1.65). All participants were right-handed, had normal or corrected-to-normal vision, and reported no history of psychiatric or neurological disorders; all provided electronic informed consent prior to participation. The study protocol was approved by the Ethics Committee of the institution where the first author belongs.

#### 2.1.2 Design and procedure

The experiment employed a 2 (economic scarcity: scarcity vs. control) × 4 (distribution: 90:10, 80:20, 70:30, 60:40) within-subjects design. Half of the participants first completed the scarcity condition, while the other half started with the control condition.

Participants were recruited from a university and completed the experiment individually in a quiet laboratory setting. Upon arrival, participants were seated in separate cubicles to ensure anonymity and prevent visual or verbal interaction. They were instructed not to communicate with others during the experiment, and screens were not visible to other participants.

After providing informed consent, participants received standardized instructions explaining the experimental task. They were told that they would take part in a series of decision-making tasks involving monetary allocations between other individuals. Importantly, participants were informed that they would act exclusively as third parties across all trials.

The experimental session proceeded as follows. In each condition (scarcity or control), participants first completed the scarcity manipulation task as described in Section 2.1.3. Immediately thereafter, they performed the third-party punishment task under the corresponding condition. Following the task, participants completed a brief questionnaire assessing their perceived resource adequacy.

After completing the first condition, participants proceeded to the second condition and repeated the same sequence of tasks. The order of conditions was counterbalanced across participants.

After completing both conditions, participants filled out a short questionnaire assessing demographic information and manipulation checks. Finally, participants were debriefed and received monetary compensation based on a fixed participation fee plus a performance-based bonus. To ensure incentive compatibility, one trial was randomly selected for payment, and participants received the remaining tokens from that trial, which were converted into real money at a rate of 0.1 RMB per token.

#### 2.1.3 Scarcity manipulation

Following the conceptual framework of Shah et al. (2012)—who induced scarcity by limiting participants’ opportunities (e.g., fewer guessing or shooting chances)—the present study induced scarcity through monetary constraints. Specifically, participants in the scarcity condition were informed that their baseline participation fee was 3 RMB, whereas those in the control condition received 15 RMB. In the task context, these amounts were operationalized as 30 versus 150 tokens, respectively, which served as the initial resource available to participants (Krosch and Amodio, 2014). This manipulation aimed to evoke a sense of economic scarcity by restricting available resources (Hilbert et al., 2022). To further assess the subjective experience of scarcity, participants rated the perceived adequacy of their resources on a 7-point Likert scale (1 = “not at all,” 7 = “very severe”) following each condition.

#### 2.1.4 Third-party punishment task

The study employed a dictator game with a third-party punishment paradigm (Fehr and Fischbacher, 2004; Meng et al., 2024). Each trial involved a dictator (Player A) and a recipient (Player B), who shared 100 tokens. The dictator could freely distribute, while the recipient had no right to reject the offer.

Participants were always assigned the role of a third party and made punishment decisions for each observed allocation. Consistent with standard third-party punishment paradigms, participants did not interact with real dictators or recipients. Instead, they were presented with pre-programmed (fictitious) allocation decisions. Participants were led to believe that these allocations were made by real individuals in previous sessions, and no information about the fictitious nature of these decisions was disclosed until debriefing.

To enhance ecological validity in the Chinese context, dictators and recipients were represented with common Chinese surnames [e.g., 王^*^(Wang), 李^*^(Li)]. Participants, acting as third parties, were each given 30 tokens at the start of each trial and could spend any portion of these tokens to punish the dictator. Each token spent reduced the dictator’s payoff by three tokens (i.e., a 1:3 ratio).

Distributions included 90:10, 80:20, 70:30, 60:40, and 50:50, with the fair 50:50 condition included but excluded from analyses, as it consistently elicited negligible punishment (Liu et al., 2022). Each condition was repeated nine times, resulting in 45 pseudo-randomized trials. Five practice trials were administered before the main task (Feng et al., 2022).

Each trial began with a fixation cross (500 ms), followed by the dictator’s distribution outcome (1,500 ms). Participants then chose whether and how much to punish the dictator. No explicit time limit was imposed on punishment decisions, allowing participants to respond at their own pace. Each trial was independent, and new fictitious players were introduced on each trial (Liu et al., 2022).

#### 2.1.5 Results

##### 2.1.5.1 Manipulation check

A paired-samples t-test confirmed the effectiveness of the scarcity induction. Participants reported significantly lower resource adequacy in the scarcity condition (*M* = 3.47, *SD* = 1.46) compared to the control condition (*M* = 5.21, *SD* = 1.28), *t* (69) = −10.05, *p* < 0.001.

##### 2.1.5.2 Robustness check

To address potential learning or carryover effects, we conducted a robustness check using only participants’ behavior in the first condition they experienced. A mixed ANOVA revealed a significant main effect of distribution, *F*(3, 33) = 55.19, *p* < 0.001, indicating that punishment increased with the level of unfairness. However, the main effect of condition (scarcity vs. control) and the interaction were not statistically significant (*ps* > 0.05).

Descriptively, the pattern of means was consistent with the main analysis, with participants in the scarcity condition showing lower levels of punishment than those in the control condition. Although the effects did not reach statistical significance, this pattern was in the same direction as the main within-subject results and may be attributable, at least in part, to reduced statistical power associated with the smaller subsamples. Therefore, these results should be interpreted with caution, but they do not contradict the primary findings.

##### 2.1.5.3 Third-party punishment

Trials with fair allocations (50:50) were excluded as punishment was virtually absent (*M* = 0; Liu et al., 2022). A 2 × 4 repeated-measures ANOVA revealed a significant main effect of distribution, *F*(3, 67) = 74.56, *p* < 0.001, *η*^2^ = 0.77: as distribution became more unfair, punishment expenditures increased Table 1.

**Table 1:** Simple effects analysis.

Scarcity condition	Distribution		Mean difference	SD	*p*	95% CI	
						Lower bound	Upper bound
Scarcity	60:40	70:30	−3.76	0.39	0.000	−4.536	−2.981
		80:20	−6.52	0.58	0.000	−7.682	−5.359
		90:10	−10.95	0.83	0.000	−12.607	−9.291
	70:30	80:20	−2.76	0.36	0.000	−3.477	−2.047
		90:10	−7.19	0.67	0.000	−8.522	−5.859
	80:20	90:10	−4.43	0.47	0.000	−5.370	−3.487
Control	60:40	70:30	−4.46	0.42	0.000	−5.305	−3.623
		80:20	−8.00	0.64	0.000	−9.277	−6.714
		90:10	−12.96	0.88	0.000	−14.714	−11.197
	70:30	80:20	−3.53	0.42	0.000	−4.369	−2.695
		90:10	−8.49	0.66	0.000	−9.814	−7.170
	80:20	90:10	−4.96	0.39	0.000	−5.745	−4.175

The main effect of economic scarcity was also significant, *F*(1, 69) = 14.93, *p* < 0.001, *η*^2^ = 0.18, indicating lower punishment expenditures in the scarcity condition (*M* = 7.00) relative to the control condition (*M* = 8.59). The interaction effect was also significant, *F*(3, 67) = 3.67, *p* < 0.05, *η*^2^ = 0.14.

**Figure 1:**
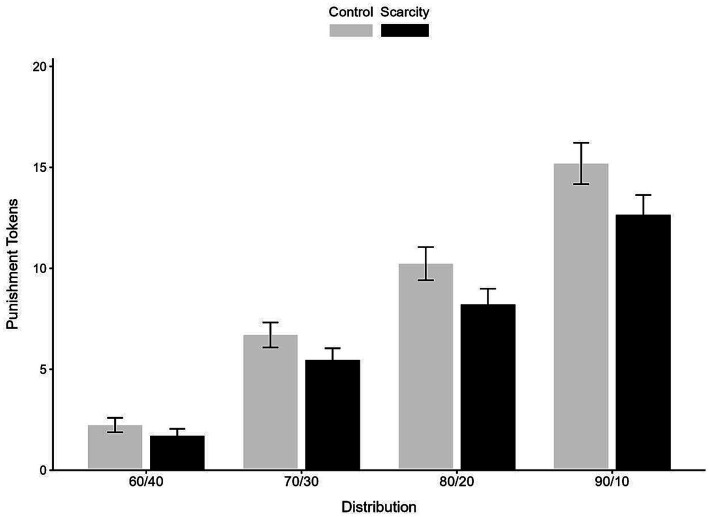
Comparison the punishment expenditures on scarcity condition.

As can be seen from Figure 1, under every distribution condition, the punishment level was lower in the scarcity treatment. Furthermore, the difference in punishment levels between the control and scarcity treatments increases with inequality in the judged distribution.

Simple effects analyses were conducted to examine the effect of distribution at each level of scarcity. Bonferroni corrections were applied to control for multiple comparisons. In the control condition, all pairwise comparisons between distribution levels were significant (*ps* < 0.001), with punishment expenditures increasing progressively as unfairness increased (all *p* < 0.001). Similarly, in the scarcity condition, all pairwise comparisons were also significant (*ps* < 0.001), showing the same pattern of increasing punishment with greater unfairness. However, the magnitude of punishment was consistently lower in the scarcity condition across all unfair distribution levels, supporting the main effect of scarcity.

#### 2.1.6 Discussion

The findings of Experiment 1 provide initial evidence that perceived economic scarcity attenuates third-party punishment, consistent with the prediction of H1. Consistent with the scarcity mindset framework (Shah et al., 2015), participants in the scarcity condition allocated fewer tokens to penalize norm violators compared to those in the control condition. Although participants had sufficient tokens to punish in both conditions, scarcity may have increased the subjective cost of each token, thereby reducing willingness rather than ability to punish. Crucially, however, a significant interaction revealed that this dampening effect of scarcity was present across all unfair distributions, yet its magnitude varied with the degree of inequity. This suggests that while scarcity exerts a general suppressive influence on norm-enforcing behavior, it does not completely override people’s sensitivity to distributional norms; rather, the absolute level of punishment remains responsive to the violation severity. These results extend prior work linking scarcity to reduced prosociality by showing its influence in the specific context of norm maintenance (Cui et al., 2022; Roux et al., 2015). However, it remains unclear whether this inhibitory effect will weaken, persist, or even intensify over time. To address this issue, Experiment 2 will further examine whether the role of economic scarcity in regulating enforcement behavior has temporal stability by comparing the impact of economic scarcity on third-party punishment behavior two months before and after, thereby providing a more comprehensive assessment of the temporal dynamics of the scarcity effect.

### 2.2 Experiment 2

#### 2.2.1 Participants

An *a priori* power analysis was conducted in G*Power 3.1. For the repeated-measures omnibus test with 16 within-subject conditions (2 × 2 × 4), we assumed a medium effect size (*f* = 0.25), *α* = 0.05, desired power = 0.80, correlation among repeated measures *r* = 0.50, and nonsphericity correction *ε* = 1. This analysis yielded a required sample size of *N* = 11. We therefore recruited *N* = 41 participants (29 females; *M* = 20.61 years, *SD* = 1.82), exceeding this target. We note that this power analysis pertains to the omnibus repeated-measures effect. As such, statistical power for detecting more complex higher-order interactions (e.g., the three-way interaction) may be comparatively lower. All participants met the same inclusion criteria as in Experiment 1 and provided electronic informed consent.

#### 2.2.2 Design and procedure

Experiment 2 employed a 2 (time: Time 1 vs. Time 2) × 2 (economic scarcity: scarcity vs. control) × 4 (distribution: 90:10, 80:20, 70:30, 60:40) within-subjects design.

In the immediate session (T1), all participants first underwent the scarcity induction (3 RMB = 30 tokens) followed by the control induction (15 RMB = 150 tokens). After each induction, participants completed the third-party punishment task identical to Experiment 1. Two months later, the same participants returned for the two-month delayed session (T2), in which the order of conditions was reversed (control first, then scarcity). This counterbalanced order across sessions ensured that potential order effects were not confounded with time. Stimuli (Chinese surnames) and task procedures were identical to those in Experiment 1.

#### 2.2.3 Results

##### 2.2.3.1 Manipulation check

A paired-samples t-test indicated that perceived resource adequacy was significantly lower in the scarcity condition than in the control condition at both time points. At T1, participants reported lower perceived resource sufficiency in the scarcity condition (*M* = 3.12, *SD* = 1.08) compared with the control condition (*M* = 5.59, *SD* = 0.95), *t*(40) = −11.43, *p* < 0.001. At T2, with perceived resource sufficiency remained lower in the scarcity condition (*M* = 3.12, *SD* = 1.14) compared with the control condition (*M* = 5.51, *SD* = 1.12), *t*(40) = −11.94, *p* < 0.001. These findings indicate that the scarcity manipulation was effective at both time points.

##### 2.2.3.2 Third-party punishment

A 2 × 2 × 4 repeated-measures ANOVA was conducted. Results replicated those of Experiment 1: distribution had a significant main effect, with more unequal allocations eliciting greater punishment, *F*(3, 38) = 51.11, *p* < 0.001, *η*^2^ = 0.80. The main effect of economic scarcity was also significant, indicating consistently lower punishment under scarcity compared to control, *F*(1, 40) = 25.44, *p* < 0.001, *η*^2^ = 0.49. Additionally, the main effect of time is not statistically significant, *F*(1, 40) = 4.14, *p* = 0.05, *η*^2^ = 0.09, indicating that punishment levels did not differ significantly between Time 1 and Time 2 Table 2.

**Table 2:** Simple effects analysis (2-month follow-up).

Scarcity condition	Distribution		Mean difference	SD	*p*	95% CI	
						Lower bound	Upper bound
Scarcity	60:40	70:30	−4.02	0.51	0.000	−5.046	−2.993
		80:20	−7.89	0.82	0.000	−9.540	−6.236
		90:10	−12.43	1.10	0.000	−14.651	−10.204
	70:30	80:20	−3.87	0.48	0.000	−4.834	−2.903
		90:10	−8.41	0.78	0.000	−9.991	−6.824
	80:20	90:10	−4.54	0.50	0.000	−5.546	−3.533
Control	60:40	70:30	−5.10	0.51	0.000	−6.129	−4.069
		80:20	−9.33	0.83	0.000	−11.005	−7.662
		90:10	−14.20	1.07	0.000	−16.354	−12.041
	70:30	80:20	−4.23	0.50	0.000	−5.249	−3.220
		90:10	−9.10	0.75	0.000	−10.619	−7.579
	80:20	90:10	−4.86	0.66	0.000	−6.189	−3.540

**Figure 2:**
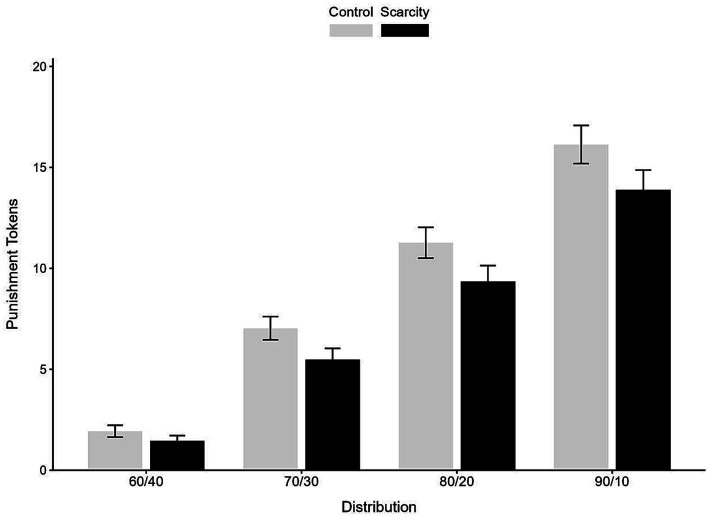
Delayed effects of scarcity and distribution on punishment expenditures (2-month follow-up).

Regarding interactions, the two-way interaction between distribution × economic scarcity was significant, *F*(3, 38) = 4.60, *p* < 0.01, *η*^2^ = 0.27. This interaction indicates that the suppressing effect of economic scarcity increased as unfairness became more severe. Specifically, the difference in punishment between the scarcity and control conditions was relatively small for mildly unequal distributions (e.g., 60:40), but became progressively larger for more unequal distributions (e.g., 80:20 and 90:10) (see Figure 2). In contrast, the interactions time × economic scarcity (*p* = 0.50) and time × distribution (*p* = 0.87) were not significant. The three-way interaction (time × economic scarcity × distribution) was not significant (*p* = 0.95), suggesting that the interaction pattern did not differ across time points. This null effect should be interpreted with appropriate caution when considering higher-order interactions.

#### 2.2.4 Discussion

Experiment 2 replicated and extended the findings of Experiment 1 by demonstrating that the suppressive effect of economic scarcity on third-party punishment is not merely situational but appears consistent across time points. Participants consistently showed a reduced willingness to punish unfair dictators under conditions of scarcity, both immediately and after a two-month delay, even when the order of scarcity and control conditions was counterbalanced. This design allows us to disentangle temporal stability from mere repetition or order effects. Although no time-related interactions were observed, conclusions regarding temporal stability should be interpreted cautiously. Importantly, a significant interaction between distribution and economic scarcity indicated that the magnitude of the suppressive effect varied as a function of unfairness. Specifically, although punishment levels were consistently lower under scarcity (reflecting a main effect), the difference between scarcity and control conditions increased as allocations became more unequal. These findings suggest that scarcity not only reduces overall punishment levels but also amplifies its suppressive effect under more severe norm violations. At the same time, punishment still increased with unfairness under both conditions, indicating that participants remained sensitive to relative inequity despite the influence of scarcity (Li et al., 2025; Zhang et al., 2025).

## 3 General discussion

This study examined the influence of economic scarcity on third-party punishment, a central mechanism for enforcing social norms. Across two experiments, scarcity consistently reduced individuals’ willingness to sanction norm violators, with effects observed both immediately and after a two-month interval. These findings extend prior research on scarcity and prosocial behavior by demonstrating its impact on behaviors essential for sustaining fairness and social order.

### 3.1 Effect of scarcity on third-party punishment

Experiment 1 demonstrated that participants exposed to scarcity allocated fewer tokens to punish unfair offers than controls. While this dampening effect was observed across all unfair allocation levels, its magnitude varied with the severity of the violation, as indicated by a significant interaction between scarcity and offer fairness. This pattern suggests that scarcity broadly suppresses norm-enforcing behavior, yet does not completely override people’s sensitivity to the degree of norm violation. One plausible explanation, consistent with prior literature, is that scarcity may narrows attentional focus and heightens concern for personal resources (Laforet et al., 2024; Van Der Veer et al., 2024), which could, in part, reflect both psychological responses to perceived scarcity and increased sensitivity to resource costs, thereby reducing willingness to incur costs for norm enforcement (David et al., 2017; Gromet and Darley, 2009). In addition, heightened perceptions of competition for limited resources may promote self-protective priorities at the expense of social obligations.

These results are consistent with prior research linking scarcity to diminished prosociality, reduced empathy, and lower cooperative engagement (Cui et al., 2022; Li et al., 2023; Roux et al., 2015), and extend these effects to third-party punishment, a critical mechanism for maintaining social norms. Notably, the significant interaction between scarcity and offer fairness clarifies that while scarcity consistently reduces punishment, its impact is modulated by the severity of the violation. This nuance underscores how resource scarcity can systematically alter third-party punishment enforcement motivation (Ahl et al., 2024; Laforet et al., 2024), offering new insight into how environmental constraints influence moral behavior beyond situational or individual differences.

### 3.2 Short-term reproducibility of scarcity effects

Experiment 2 examined whether the attenuating effect of scarcity on third-party punishment is consistent over time. Consistent with Experiment 1, participants exhibited reduced third-party punishment under scarcity at both the immediate and two-month assessments. This consistent pattern of results across time, achieved with a counterbalanced design that rules out order effects, provides support for the consistency of this effect across time points on punishment. However, further research with larger samples would be valuable to more rigorously assess temporal stability.

Combining the results of Experiment 1 and Experiment 2, this study provides more reliable evidence for the influence of scarcity on third-party punitive behavior. Firstly, the scarcity effect is robust, meaning that a consistent weakening effect can be observed in experiments involving different time points and manipulation sequences. Secondly, the scarcity effect demonstrates consistency across time points and measurement occasions. Even 2 months after the manipulation interval, the scarcity condition can still trigger similar behavioral patterns. Finally, the impact of economic scarcity on third-party penalties is mainly reflected in reducing the overall level of resource input rather than changing the judgment of the degree of normative violations or the sensitivity to fairness.

These findings have significant social significance. Economic scarcity not only weakens individuals’ willingness to follow norms in the short term, but may also weaken the mechanism for punishing violations over a longer time scale, and the punishment mechanism is a key link in maintaining social cooperation and order. These findings raise the possibility that prolonged or widespread perceptions of resource scarcity could attenuate norm-enforcing behaviors in broader social contexts, potentially affecting cooperative dynamics over time. However, caution is warranted in extrapolating beyond the laboratory setting. The results of Experiment 2 emphasize that scarcity is not only an objective resource condition but also associated with subjective and behavioral adjustments that may influence social decision-making over time. Its potential social cost deserves further attention.

Overall, the present findings position economic scarcity as a significant psychosocial determinant of third-party punishment. By demonstrating both immediate and enduring effects, the study indicates that perceived resource insufficiency and associated resource constraints can broadly suppress prosocial enforcement behaviors, extending prior research on generosity, cooperation, and sharing. Importantly, scarcity tends to reduces the willingness to invest resources in punishment, without eliminating the relative differentiation of unfair offers, indicating that perceived scarcity primarily affects willingness to punish rather than fairness sensitivity. These insights refine theoretical models of moral and prosocial decision-making by integrating environmental and motivational influences. They further demonstrate that scarcity systematically biases social behavior, even under controlled experimental conditions.

### 3.3 Implications

The present study provides both theoretical and practical insights. Theoretically, it extends the scarcity mindset framework by showing that economic scarcity inhibits third-party punishment, highlighting scarcity’s broader impact on mechanisms that sustain social norms. The temporal stability observed in Experiment 2 suggests that scarcity may function as a relatively persistent psychological influence across short-term intervals.

Practically, these findings indicate that perceived scarcity may reduce individuals’ engagement in sanctioning unfairness, thereby undermining social cooperation and enabling norm violations to persist. Interventions that mitigate scarcity perceptions, foster empathy, or provide structured avenues for norm enforcement may help maintain cooperative behavior. Policymakers and organizations should consider these psychological dynamics when promoting prosocial actions and designing programs to sustain social order in resource-constrained or unequal contexts.

### 3.4 Limitations and future directions

While these findings carry important theoretical and practical implications, several limitations should be acknowledged. First, scarcity was manipulated via differences in monetary endowment, which may not fully capture real-world economic constraints (e.g., chronic financial hardship or time scarcity) and may also alter the relative cost of punishment. As such, the observed reduction in punishment may partly reflect a standard income effect in addition to psychological scarcity. Future research could enhance ecological validity while disentangling these influences by employing more realistic scarcity manipulations or by independently varying perceived scarcity and objective resource levels. Second, the sample consisted mainly of undergraduate students, limiting applicability to other age groups, socioeconomic backgrounds, or cultural contexts; subsequent studies should examine diverse populations. Third, the underlying psychological mechanisms were not directly measured. Although the present study included a subjective assessment of perceived resource adequacy as a manipulation check, this proximal psychological state was not incorporated into the main analytical framework. Future research should more systematically examine perceived resource adequacy, as well as other related processes (e.g., attentional focus, empathy, or stress), as potential mediating mechanisms to clarify how economic scarcity influences third-party punishment. Fourth, although Experiment 2 demonstrated temporal stability over 2 months, longer-term effects remain unclear, highlighting the need for extended longitudinal studies to assess enduring impacts on norm enforcement.

## 4 Conclusion

Across two experiments, the present study consistently found that economic scarcity was associated with reduced engagement in third-party punishment. Participants allocated fewer resources to sanction unfair allocators under scarcity compared to control conditions.

## Data Availability

The raw data supporting the conclusions of this article will be made available by the authors, without undue reservation.
